# Anti-Inflammatory Effects of *Lactobacillus Rahmnosus* and *Bifidobacterium Breve* on Cigarette Smoke Activated Human Macrophages

**DOI:** 10.1371/journal.pone.0136455

**Published:** 2015-08-28

**Authors:** Esmaeil Mortaz, Ian M. Adcock, Fabio L. M. Ricciardolo, Mohammad Varahram, Hamidreza Jamaati, Ali Akbar Velayati, Gert Folkerts, Johan Garssen

**Affiliations:** 1 Cell and Molecular Biology Group, Airways Disease Section, National Heart and Lung Institute, Faculty of Medicine, Imperial College London, Dovehouse Street, London, United Kingdom; 2 Chronic respiratory research center, National Research and Institute of Tuberculosis and Lung Diseases (NRITLD), Shahid Beheshti University of Medical Sciences, Tehran, Iran; 3 Department of Immunology, Faculty of Medicine, Shahid Beheshti University of Medical Sciences, Tehran, Iran; 4 Department of Clinical and Biological Sciences, University of Torino, Torino, Italy; 5 Mycobacteriology Research Center (MRC) National Research Institute of Tuberculosis and lung diseases (NRITLD), Shahid Beheshti University of Medical Sciences, Tehran, Iran; 6 Division of Pharmacology, Utrecht Institute for Pharmaceutical Sciences, Faculty of Science, Utrecht University, Utrecht, The Netherlands; 7 Nutricia Research Centre for Specialized Nutrition, Utrecht, The Netherlands; University of Rochester Medical Center, UNITED STATES

## Abstract

**Background:**

Chronic obstructive pulmonary disease (COPD) is a major global health problem with cigarette smoke (CS) as the main risk factor for its development. Airway inflammation in COPD involves the increased expression of inflammatory mediators such as CXCL-8 and IL-1β which are important mediators for neutrophil recruitment. Macrophages are an important source of these mediators in COPD. *Lactobacillus rhamnosus* (*L*. *rhamnosus*) and *Befidobacterium breve* (*B*. *breve*) attenuate the development of ‘allergic asthma’ in animals but their effects in COPD are unknown.

**Objective:**

To determine the anti-inflammatory effects of *L*. *rhamnosus* and *B*. *breve* on CS and Toll-like receptor (TLR) activation.

**Design:**

We stimulated the human macrophage cell line THP-1 with CS extract in the presence and absence of *L*. *rhamnosus* and *B*. *breve* and measured the expression and release of inflammatory mediators by RT-qPCR and ELISA respectively. An activity assay and Western blotting were used to examine NF-κB activation.

**Results:**

Both *L*. *rhamnosus* and *B*. *breve* were efficiently phagocytized by human macrophages. *L*. *rhamnosus* and *B*. *breve* significantly suppressed the ability of CS to induce the expression of IL-1β, IL-6, IL-10, IL-23, TNFα, CXCL-8 and HMGB1 release (all p<0.05) in human THP-1 macrophages. Similar suppression of TLR4- and TLR9-induced CXCL8 expression was also observed (p<0.05). The effect of *L*. *rhamnosus* and *B*. *breve* on inflammatory mediator release was associated with the suppression of CS-induced NF-κB activation (p<0.05).

**Conclusions:**

This data indicate that these probiotics may be useful anti-inflammatory agents in CS-associated disease such as COPD.

## Introduction

COPD is currently the 4th biggest killer worldwide and is expected to be the third leading cause of death over the next 10 years [1]. Smoking is the most important lifestyle risk factor for pathogenesis of COPD [2], the presence of which also enhances the risk of developing lung cancer [3]. The level of inflammation in the airways of COPD patients correlates strongly with disease severity and is critical to the development and progression of disease [4–6]. Attention has traditionally centered on the role of macrophages and neutrophils in disease development [7]. Macrophages synthesize and secrete many mediators which play a role in COPD inflammation and innate immune responses to potentially pathogenic organisms [8].

The chronic inflammation in COPD shares many epidemiological, inflammatory and immune characteristics with other chronic diseases such as inflammatory bowel disease (IBD) [9]. The crosstalk between the pulmonary and intestinal mucosal in COPD and IBD has recently been extensively reviewed [10–14]. The concept that probiotics confer health benefits has gained much attention [15]. Most probiotics contain virulent lactic acid-producing bacteria (Lactobacillus, Streptococcus, Bifidobacterium, and Enterococcus) or non-pathogenic yeasts such as Saccharomyces boulardii [16, 17], and have been advocated for the prevention and treatment of various conditions, including gastroenteritis, clostridium-associated diarrhea, inflammatory bowel disease, food allergies, and dental cavities [18–21]. With the exception of treatment of infectious diarrhea in both adults and children [19 22], the evidence of their effectiveness remains inconclusive [23, 24].

Administration of lactobacilli orally may not only modulate local inflammation [25], but might also act systemically and have effects on other organs and tissues [26]. The probiotic hypothesis proposes that perturbations in the gut microbiota resulting from antibiotic use and dietary differences can disrupt the normal mechanisms of immunologic tolerance in the gut mucosa leading to an increase in the incidence of allergic disease, including asthma [27] and viral infections [28,29]. Sagar et al demonstrated that the combination of *Bifidobacterium breve (B*. *breve)* with non-digestible oligosaccharides suppresses airway inflammation in a murine model for chronic asthma [30], which could explain the link of gut and respiratory system.

Macrophages within the airway are derived from blood monocytes that track into the lung following migration signals and are a major cell type in the pathogenesis of COPD [5,7,8]. Peripheral blood monocytes and monocyte-derived-macrophages from COPD patients have defective cellular functions and this may reflect signals derived from other organs including the gut as these cells traffic around the body [5,7,8]. It is possible, therefore, that probiotic signals may directly target blood monocytes as they traffic through the gut.

Studies have also shown that probiotics such as *L*. *reuteri* inhibit TNF-αinduced CXCL-8 expression in intestinal epithelial cells [31] and the release of proinflammatory cytokines by human macrophages via inhibition of c-Jun pathways [32]. Although the mechanism of action of these beneficial bacteria still needs to be elucidated, probiotics have been shown to contain Toll- like receptor (TLR) ligands and can thereby attenuate TLR-driven Th1 responses [33]. TLRs and NOD-like receptors (NLRs) are key pattern recognition receptor (PRR) families in the innate immune response, which are also involved in the activation and shaping of adaptive immunity [34].

There is no data examining the effect of probiotics on cigarette smoke-induced inflammation in macrophages despite these being key cells in the pathogenesis of COPD and are activated by TLRs. In the current study we investigated the mechanisms by which *Lactobacillus rhamnosus (L*. *rhamnosus)* and *B*. *breve* modulate cigarette smoke-induced inflammatory mediator expression in human macrophages.

## Materials and Methods

### Cell culture and reagents

The human monocytic cell line, THP-1 (American Type Culture Collection, Manassas, VA), was maintained in suspension culture in RPMI-1640 medium supplemented with 2% (v/v) penicillin-streptomycin (Pen-Strep), 36μM N-2-hydroxethyl-piperazine-N'-2-ethanesulfonic acid (HEPES) (Invitrogen Life Technologies, Burlington, ON, Canada), and 10% (wt/v) fetal bovine serum (FBS) (CanSera, Toronto, ON, Canada). For differentiation into a macrophage-like phenotype, THP-1 cells were seeded onto six-well sterile plastic culture plates (VWR, Mississauga, ON, Canada) at 250,000 cells/well (unless stated otherwise) and treated with 10nM PMA (Sigma, St. Louis, MO) as described previously [35]. The media was replaced with PMA-free medium 72h later and the experimental conditions established after an additional 24h of culture (see below). Macrophage differentiation was monitored by morphology, FACS and cell adherence to plastic and >90% of THP-1 cells had a macrophage phenotype. Monocytic U937 cells (ATCC) were cultured in full RPMI 1640 medium (containing 1% L-glutamine and 10% FCS; Invitrogen, Paisley, UK) and serum starved in minimal RPMI 1640 medium (1% L-glutamine and 0.5% FCS) overnight before treatment.

### Preparation of cigarette smoke extracts (CSE)

CSE was prepared as described previously [36]. Briefly, CSE was generated by the burning of commercially available Lucky Strike cigarettes without filter (British–American Tobacco, Groningen, The Netherlands), using the TE-10z smoking machine (Teague Enterprises, Davis, CA, USA) which is programmed to smoke cigarettes according to the Federal Trade Commission protocol (35ml puff volume drawn for 2s/min). The machine produced main and side stream smoke from one cigarette through 5ml RPMI without phenol red. The absorbance was then measured using a spectophotometer and the media were standardized to a standard curve of CSM concentration against absorbance at 320nm. The pH of the resultant extract was titrated to pH 7.4 and diluted with RPMI medium. This concentration (OD = 4.0) was serially diluted with untreated media to 0.03, 0.06 and 0.12 OD and applied to the cells.

### FITC labeling of bacterial strains and phagocytosis assays

Bifidobacterium breve M-16 V (B. breve, Morinaga Milk Industry, Tokyo, Japan) and Lactobacillus rhamnosus NutRes1 (L. rhamnosus, Danone Research, Wageningen, the Netherlands) were grown in MRS (Oxoid, Basingstoke, UK), supplemented with 0.5g/L L-cysteine for Bifidobacteria, at pH 6.5 and under anaerobic conditions. Bacteria were harvested in the early stationary phase, washed with phosphate buffered saline (PBS, Lonza Leusden, The Netherlands) and stored in 20% glycerol in aliquots at—80°C as described earlier [37, 38].

Bacterial cells were harvested from exponential phase cultures, washed three times in PBS and resuspended in appropriate media at 2×10^8^ bacteria/ml and then labeled with FITC as described previously [39]. Briefly, bacteria were heat killed for 1h at 70°C, centrifuged (16000 xg for 5 min), washed three times in carbonate buffer, pH 9.4 and re-suspended in the same buffer. FITC was added to a final concentration of 0.15mg/ml and the bacteria were incubated for 30min at room temperature on a rotary mixer. Cells were then washed three times in carbonate buffer to remove all traces of free FITC, adjusted to 2×10^8^/ml in PBS and stored at −20°C until used. For phagocytosis assays THP-1 cells were incubated with FITC-conjugated *L*. *rhamnosus* and *B*. *breve* (MOI 1:6) at 37°C or at 4°C for 2h and before washing with 4x cold PBS and then centrifuged at 100 x*g*. The percentage of cells that bound FITC-conjugated bacteria was measured by flow cytometry (40,000 events). FITC-Dextran (Sigma-Aldrich, USA) was used as a positive control. Phagocytosis was determined by comparing the intensity of green fluorescence (FITC) before and after trypan blue quenching of membrane-bound, labeled bacteria.

### THP-1 cell stimulation

THP-1 cells were grown in RPMI 1640 medium supplemented with 10% FBS, 2mM L-glutanine, 100U/mL penicillin and 100g/mL streptomycin (RPMI medium) at 37°C in 5% CO_2_, 95% air in the presence of CSE for 24h. *L*. *rhamnosus* (NutRes 1 formerly known as NumRes 1) and *B*. *breve* (NutRes 204 formerly known as NumRes 204) were provided by Danone Research BV (Wageningen, the Netherlands) as live bacteria in a 20% glycerol stock having been grown as previously described [38].

THP-1 cells (1x10^6/^ml) were inoculated at a density of 5×10^5^/ml with 3 ratios of *L*. *rhamnosus and B*. *breve* (1:10, 1:20 and 1:50) for 2h prior to exposure with CSE (0.06 OD) for 16h. Supernatants were harvested and stored at -20°C until mediator release was determined by ELISA. For mRNA detection, cells (5x10^6^ cells/per experiment) were stimulated for 3h; for NF-κB/SEAP activity cells (1x10^6^ cells/experiment) were activated for 24h and for p65 translocation experiments cells (3x10^6^ cells/per experiment) were activated for 30 min.

### Real-time quantitative PCR

Total RNA was extracted using High Pure RNA Isolation Kit (Roche Applied Science, USA) according to the manufacturer's instructions. The quantity and purity of the RNA was determined by Nanodrop (Nanodrop Tec, Wilmington, DE, USA) and the ratio of 260/280 nm of all the samples was higher than 2. Equal amounts of total RNA was reverse-transcribed using Transcriptor first strand cDNA synthesis kit (Roche) using oligo(dT). Real-time PCR was performed using SYBR Green PCR Master-Mix (ABGene) in 20μl reactions with 0.5μM primers for 40 cycles on an ABI Prism 7000 sequence detector (Applied Biosystems). PCR conditions were 50°C for 2min and 95°C for 15min, followed by 40 cycles of 95°C for 15s and 60°C for 1min. Primers were designed using the Primer beb3 software which are as follows:

*CXCL-8*, Forward 5′-AACAGGTGCAGTTTTGCCAAG-3′;

Reverse 5′-CGCAGTGTGGTCCACTCTCA-3′ and

GAPDH, Forward 5′-CCAGGTGGTCTCCTCTGACTTC-3′;

Reverse 5′-CACCCTGTTGCTGTAGCCAAA-3′.

The raw CTs from the reactions were analyzed by a modified delta-*C*_t_ method with efficiency correction using a PCR data analysis program, qBase to obtain relative quantification values.

### Quantification of CXCL-8, TNF-α, IL-6, IL-10, IL-1β and IL-23 production

CXCL-8 concentrations in the cell supernatant were quantified using ELISA (BD Biosciences Pharmingen, Breda, The Netherlands) according to the manufacturer's instructions. The production of other inflammatory cytokines (TNF-α, IL-6, IL-10, IL-1β and IL-23) was measured using Bio-Plex (Invitrogen) according to the manufacturer's instructions.

### Preparation of whole cell extracts and Western blot analysis

Cells were washed twice with PBS and total protein extracts were prepared using radioimmunoprecipitation assay (RIPA) buffer supplemented with protease inhibitor cocktail (MiniTM protease inhibitors, Roche Diagnostics). Samples were stored at -70°C until analysed. Protein concentrations were determined by BCA protein assay kit (Pierce) and samples (25μg) were subjected to SDS/PAGE [10% (w/v) gel] as previously described [40]. The separated proteins were electro blotted on PVDF membranes (Bio-Rad). Membranes were then washed once with Tris/HCI, pH 7.4, containing 159mM NaCI and 1% Tween 20 (TBS-T), and then blocked in super-blocking buffer (Pierce) for 1h. After washing the membranes with TBS-T, antibodies against IL-1β (Cleaved IL-1β (Asp116) Antibody #2021, Bioke, Leiden, The Netherlands) was added for 24h at 4°C. Bands were detected using 1:20,000 HRP-conjugated goat anti-rabbit IgG for IL-1β (Dako B.V. Heverlee, Belgium) and visualized with an enhanced chemiluminescence Western blot analysis system (Amersham Pharmacia Biotech). Films were scanned and analyzed on a GS7-10 Calibrated Imaging Densitometer equipped with Quantity One v. 4.0.3 software (Bio-Rad, USA). For detection of equal loaded protein on the gel, the membranes were stripped with stripping buffer (Pierce), incubated with antibody to histone H1 (Santa Cruz Biotechnology, Santa Cruz, CA), as a housekeeping protein and visualized by ECL.

### Measurement of NF-κB activity and p65 translocation

NF-κB activity was determined using the secreted embryonic alkaline phosphatase (SEAP) activity assay. Aliquots of culture medium were clarified by centrifugation at 14,000 xg for 2min, heated at 65°C for 5 min to inhibit endogenous phosphatase activities, adjusted to 1x SEAP assay buffer (0.5M carbonate, pH 9.8, 0.5mM MgCl_2_), and then incubated at 37°C for 10min in a 96-well culture dish. Fifty microliters of 6M p-nitrophenylphosphate (Sigma-Aldrich, USA) dissolved in SEAP assay buffer (pre warmed to 37°C) was added to the mixture (to a final volume of 200μl). The absorbance at 405nm (A_405_) of the reaction mixture was read in a Wallac 1420 plate reader (PerkinElmer, USA). SEAP activity is given in milliunits (mU) per ml. One milliunit is defined as the amount of phosphatase that hydrolyzes 1.0pmol of p-nitrophenylphosphate per min, and this corresponds to an increase of 0.04mU per min.

Western blot analysis was used to evaluate p65 nuclear translocation. Nuclear and cytoplasmic cell extracts were prepared using an NE-PER kit (Thermo Fisher Scientific, USA) according to the manufacturer’s instructions. Subcellular localization of p65 was detected using a rabbit anti-human p65 antibody (Santa Cruz Biotechnology, USA) and visualized as described above.

### Viability assay

Viability of cells was determined by staining cells with Annexin-V or 7-ADD by flow cytometry analysis (BD, USA).

### Statistics

Results are expressed as percent of unstimulated and-stimulated controls and are shown as means ±standard error of the mean (S.E.M.). For statistical evaluation of the data the program SPSS 18.0 was used and normally distributed data compared using ANOVA and student’s T-test. Some of the data sets were not normally distributed and group comparisons were performed using non parametric tests (Friedman and Mann–Whitney *U*-tests) and data presented as median (95% confidence intervals); *p*<0.05 was considered to indicate significance.

## Results

### Transformed THP-1 cells efficiently phagocytose bacteria

**Fig 1:**
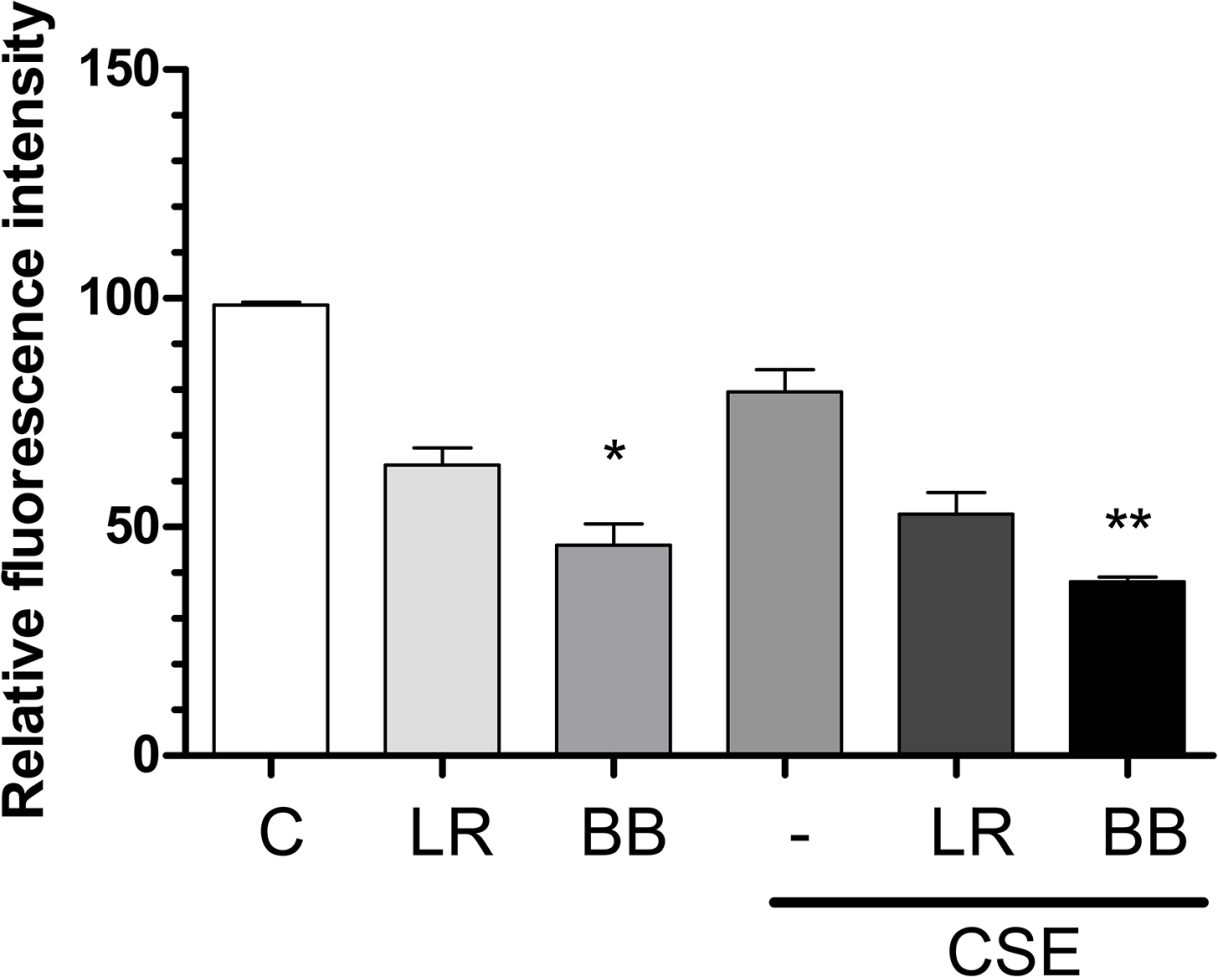
THP-1 cells efficiently phagocytose bacteria. THP-1 cells (1x10^6^) were incubated with FITC-labelled *L*. *rhamnosus* (LR) or *B*. *breve* (BB) at a ratio of 1:10 for 2h at 37°C in the presence or absence of 1.5% cigarette smoke extract (CSE). Cells were washed and the presence of intracellular label detected in 40,000 events by flow cytometry. The uptake of FITC-dextran was used as a positive control (C) and all results are presented as means ± S.E.M. of the uptake compared to this. N = 4 independent experiments, *p<0.05, **p<0.01 versus control.

We used examined the phagocytosis of FITC-labelled *L*. *rhamnosus* and *B*. *breve* (10^6^ microorganisms/well) at a ratio of 10:1 (bacteria: THP-1). A lower percentage of cells [63.0% (51.3–75.5)] took up FITC-*L*. *rahmanosus* after 2h compared with *B*. *breve* [48.0% (31.2–60.8)] compared with FITC-Dextran [98.5% (96.7–100.5)] (Table 1 and Fig 1). This was significant for *B*. *breve* (p<0.05). Inhibition of phagocytosis by cytochalasine D (10mg/ml) prevented uptake of FITC labelled bacteria (data not shown) and no phagocytosis occurred in cells cultured at 4°C (Table 1). Incubation of cells with 1.5% CSE did not significantly suppress the phagocytosis of FITC-dextran (Fig 1) or further reduced the ability of these cells to phagocytose bacteria (Fig 1).

**Table 1:** THP-1 cells efficiently phagocytose FITC-labeled-*L. rhamnosus* and *B. breve*.

Temperature	FITC-*L*. *rhamnosus*	FITC- *B*. *breve*
37°C	63.0 (51.5–75.5)	48.0 (31.2–60.8)
4°C	11.0 (8.5–13.5)	6.0 (2.5–10.1)

Data are presented as median (95% confidence intervals) of n = 4 independent experiments.

### *L*. *rhamnosus* and *B*. *breve* enhance basal CXCL-8 release but suppress CSE-induced CXCL-8 production

**Fig 2:**
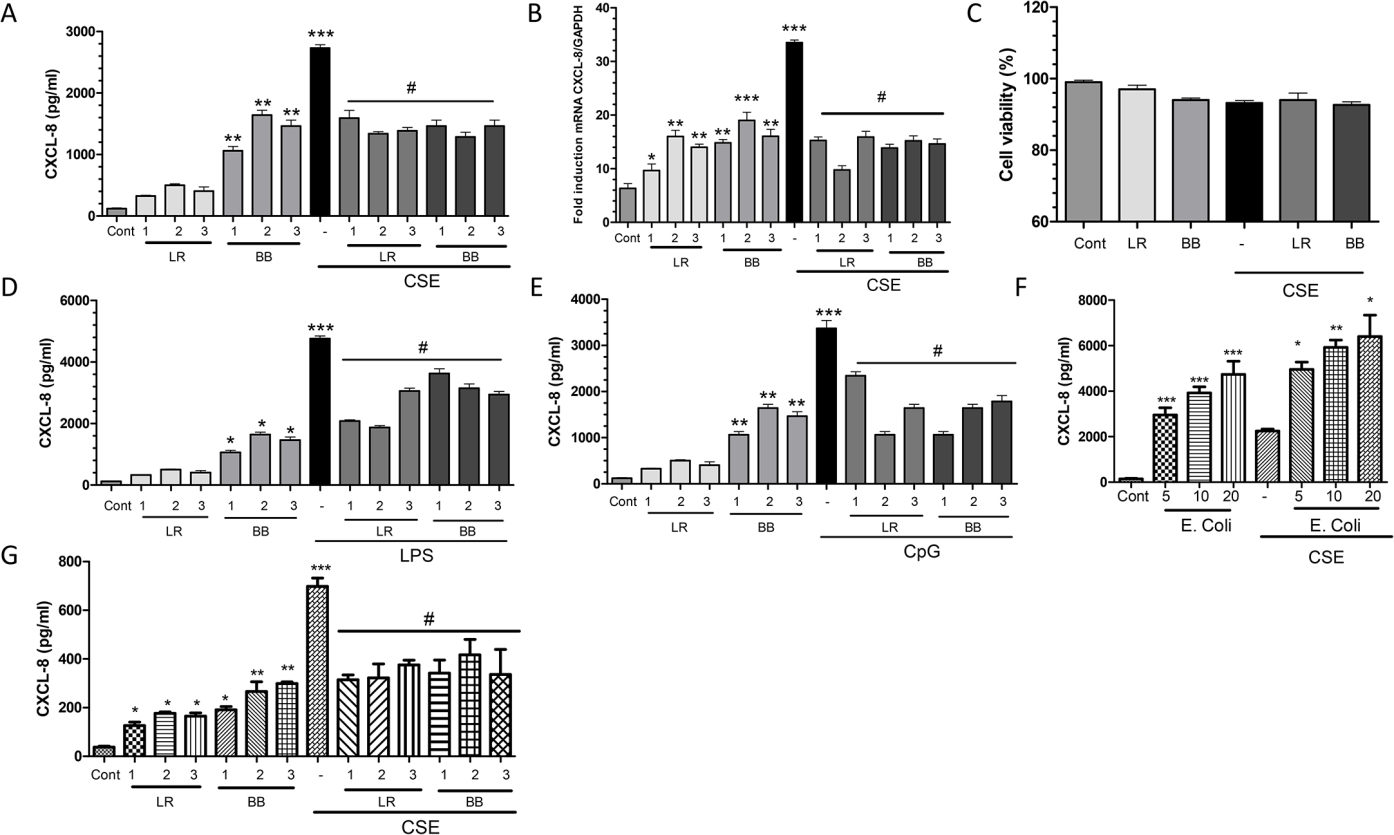
The effect of *L*. *rahmonusus (*LR*) and B*. *breve* (BB) on cigarette smoke extract (CSE)-, lipopolysaccharide (LPS)- and CpG-induced CXCL-8 release by THP-1 macrophages. Cells 1x10^6^/ml were pretreated for 2hrs with 10 bacteria/cell (1), 20 bacteria/cell (2) and 50 bacteria/cell (3) LR and BB in the presence and absence of 1.5% CSE and CXCL-8 release (A) and mRNA expression (B) determined after 16 and 3hrs respectively. Expression of CXCL-8 mRNA is expressed as a ratio of the housekeeping gene GAPDH. Cell viability as assessed by Annexin V expression was not affected by any treatment (C). A similar effect of LR and BB on (D) LPS (1000ng/ml)-and (E) CpG oligonucleotide (3μM)-stimulated CXCL-8 release at 16h was also seen. (F) Cells were preincubated for 2 h with E.coli (at various ratios of bacteria to cells) and then stimulated with 1.5% CSE and CXCL-8 release determined after 16hrs. (G) U937 macrophages were stimulated with LR and BB in the presence and absence of 1.5% CSE and CXCL-8 release determined after 16hrs. Data are presented as mean ± S.E.M. of three independent experiments. *p < 0.05; **p < 0.01 versus control and ^#^p<0.05 versus CSE-stimulated CXCL-8.

There was a dose-dependent induction of CXCL8 release from transformed THP-1 cells by *L*. *rhamnosus* and *B*. *breve* up to 20 bacteria/cell but this is not further enhanced at 50 bacteria/cell (Fig 2A). LR and BB The induction of CXCL-8 by *B*. *breve* was greater than that observed with *L*. *rhamnosus*. In all subsequent experiments, a sub-maximal ratio of 1:10 was selected.

CSE caused a concentration-dependent induction of CXCL-8 release from transformed THP-1 cells (data not shown). 1.5% CSE significantly increased the release of CXCL-8 (p<0.001, Fig 2A) and this concentration was used in all subsequent experiments.

In contrast to the effect of *L*. *rhamnosus* and *B*. *breve* on basal expression of CXCL-8, pre-treatment of cells with *L*. *rhamnosus* and *B*. *breve* attenuated CSE-induced CXCL-8 release (Fig 2A). There was no difference in the ability of *L*. *rhamnosus* and *B*. *breve* to suppress CSE-induced CXCL-8 release and no obvious dose-response was observed (Fig 2A). A similar, pattern was seen when examining the effect of *L*. *rhamnosus* and *B*. *breve* on baseline and CSE-induced CXCL-8 mRNA expression (Fig 2B). CSE and the bacteria had no effect on cell viability (Fig 2C) at a dose of 10 bacteria per cell.

A similar ability of *L*. *rhamnosus and B*. *breve* to attenuate stimulated CXCL-8 release was also seen when cells were stimulated with the TLR4 agonist LPS (1000ng/ml) (Fig 2D) and the TLR9 agonist CPG ODN (3μM) (Fig 2E). *L*. *rhamnosus* attenuated CXCL-8 release to a similar extent in LPS- and CpG-stimulated cells (55 and 50% suppression for LPS and CPG stimulation, p≤0.05 and P≤0.01 respectively) and a similar effect was seen with *B*. *breve* (50 and 65% suppression for BB with LPS and CPG stimulation, p≤0.05 and P≤0.01 respectively).

To show that probiotics but not pathogens inhibit the pro-inflammatory effects of CSE, THP-1 cells were incubated with E.coli bacteria and then stimulated with CSE for 16 h and the levels of CXCL-8 was evaluated in supernatants. In contrast to probiotics, E. coli enhanced the ability CSE to stimulate CXCL-8 release (Fig 2F).

In addition, to determine whether the effects seen were cell-type dependent, we used a second macrophage cell type U937 and conducted similar experiment. U937 macrophages were stimulated with LR and BB in the presence and absence of 1.5% CSE and CXCL-8 release determined after 16hrs. A similar response to that observed in THP-1 cells was observed (Fig 2G).

### Effect of *L*. *rhamnosus* and *B*. *breve* on IL-10, TNF-α, IL-1β, IL-6, and IL-23 release in the presence and absence of CSE

**Fig 3:**
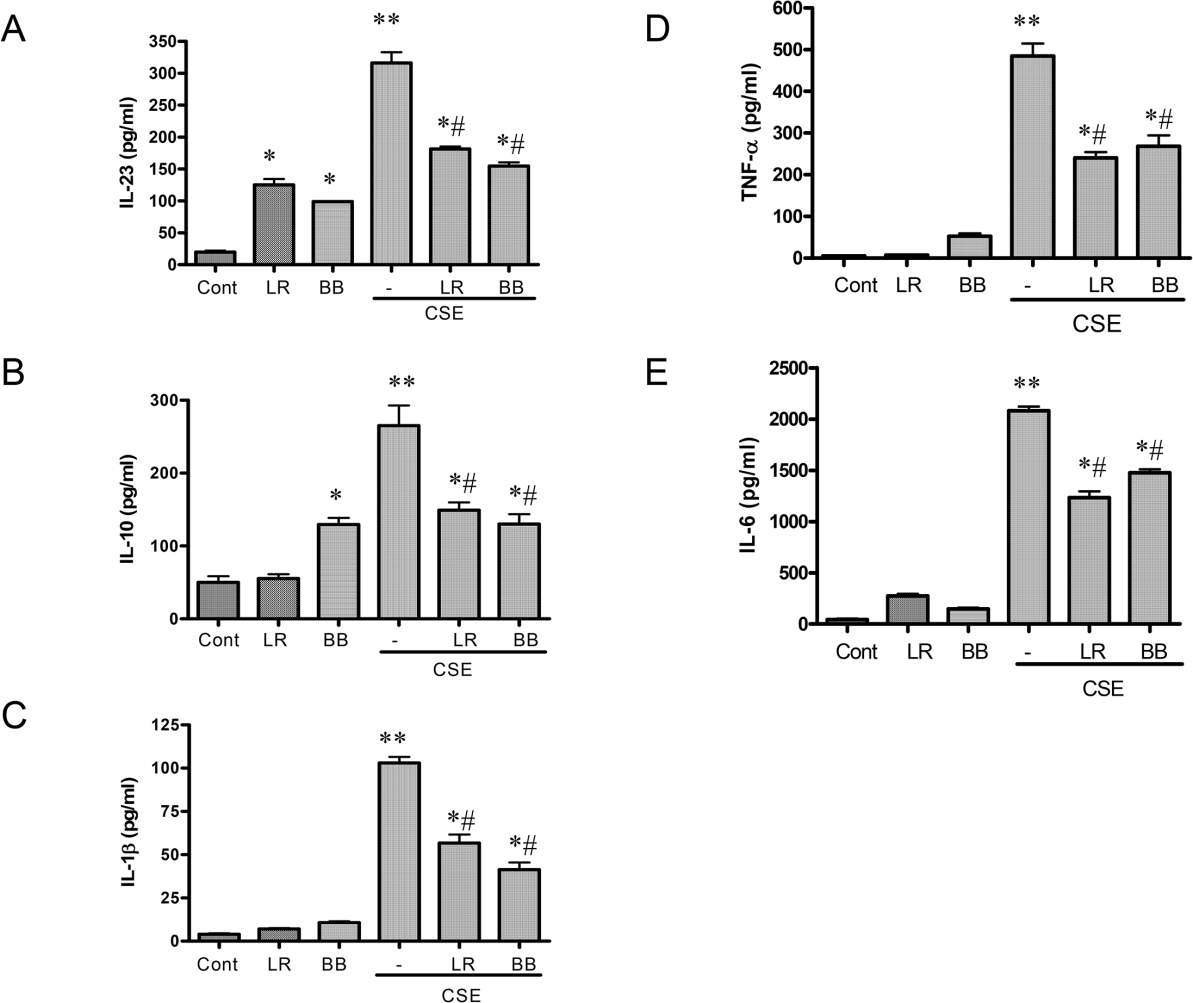
Effects of *L*. *rhamnosus* (LR) and *B*. *breve* (BB) on the release of cytokines induced by cigarette smoke extract (CSE). THP-1 cells (1x10^6^/ml) were preincubated for 2h with LR or BB (both at 10 bacteria/cell) in the presence or absence of 1.5% CSE and the release of IL-23 (A), IL-10 (B), IL-1β (C), TNF-α (D) and IL-6 (E) assessed after 16hrs. Data are presented as mean ± S.E.M. of three independent experiments. *p < 0.05; **p < 0.01 versus control and ^#^p<0.05 versus CSE-stimulated mediator release.

*L*. *rhamnosus* (1:10 bacteria:cells) significantly enhanced the expression of basal IL-23 (Fig 3A) but had no effect on the basal release of IL-10 (Fig 3B), IL-1β (Fig 3C), TNF-α (Fig 3D) or IL-6 (Fig 3E). *B*. *breve*, in contrast, at the same dilution significantly stimulated basal production of both IL-23 and IL-10 (Fig 3A & 3B) but again had no effect on basal release of IL-1β, TNFα or IL-6. Both *L*. *rhamnosus* and *B*. *breve* had a similar significant ability to suppress CSE-induced expression of IL-10, TNF-α, IL-1β, IL-6, and IL-23 by 40–50% in all cells (Fig 3A–3E). We have subsequently examined the expression of the mRNA for these cytokines in this study and show similar effects (data not shown).

### *L*. *rhamnosus* and *B*. *breve* suppress CSE-induced NF-*κ*B activity

**Fig 4:**
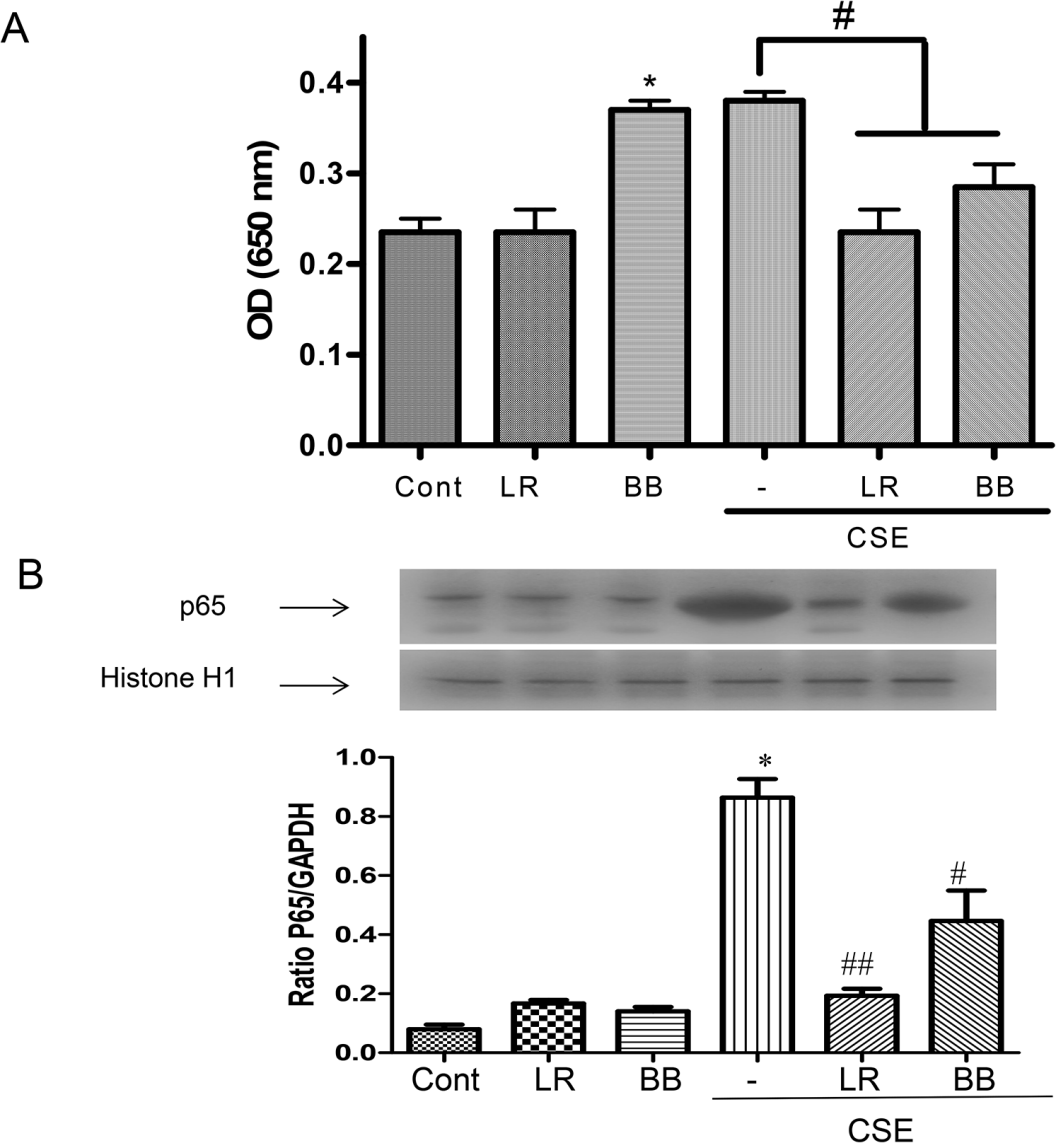
Effects of *L*. *rhamnosus* (LR) and *B*. *breve* (BB) on cigarette smoke extract (CSE)-induced NF-κB activation. Cells 5x10^4^ /well were preincubated for 2h with LR or BB (both at 10 bacteria/cell) and then incubated for 24h in the presence or absence of 1.5% CSE. SEAP activity was measured in cell culture supernatant (A). *p<0.05 compared to the control and ^#^p< 0.05 compared to CSE alone. The effect of RL and BB on NF-κB p65 nuclear import was also assessed by Western blotting with histone H1 (H1) as a nuclear loading control (B). A representative blot of 3 independent experiments is shown (upper panel) with the mean±S.E.M. data presented graphically (lower panel). *p < 0.05; **p < 0.01 versus control and ^#^p<0.05 versus CSE-stimulated p65 nuclear import.

Since CXCL-8 and many of the other inflammatory mediators up-regulated by CSE are controlled by the transcription factor NF-κB, we next analyzed whether *L*. *rhamnosus* (1:10 bacteria:cells) and *B*. *breve* (1:10 bacteria:cells) had any effect on basal and CSE-stimulated NF-κB activity as determined by SEAP assay. Pre-incubation of transformed THP-1 cells with *B*. *breve*, but not *L*. *rhamnosus*, significantly increased basal NF-κB activity (Fig 4A). CSE exposure also resulted in a similar significant induction of NF-κB activity. However, pre-incubation of cells with both *L*. *rhamnosus* and *B*. *breve* resulted in a significant reduction in CSE-stimulated NF-κB activation back to baseline levels (Fig 4A).

Next we determined nuclear p65 translocation by Western blotting as an alternative measure of NF-κB activation (Fig 4B). Neither *L*. *rahmanosus* nor *B*. *breve* had any effect on basal p65 nuclear translocation at the single 15min time point examined whereas CSE significantly increased p65 nuclear import. Consistent with the effects of *L*. *rahmanosus* and *B*. *breve* pre-incubation on NF-κB activity, CSE-enhanced p65 nuclear translocation was markedly suppressed by these bacteria (Fig 4B).

### Effects of *L*. *rhamnosus* and *B*. *breve* on IL-1β activation

**Fig 5:**
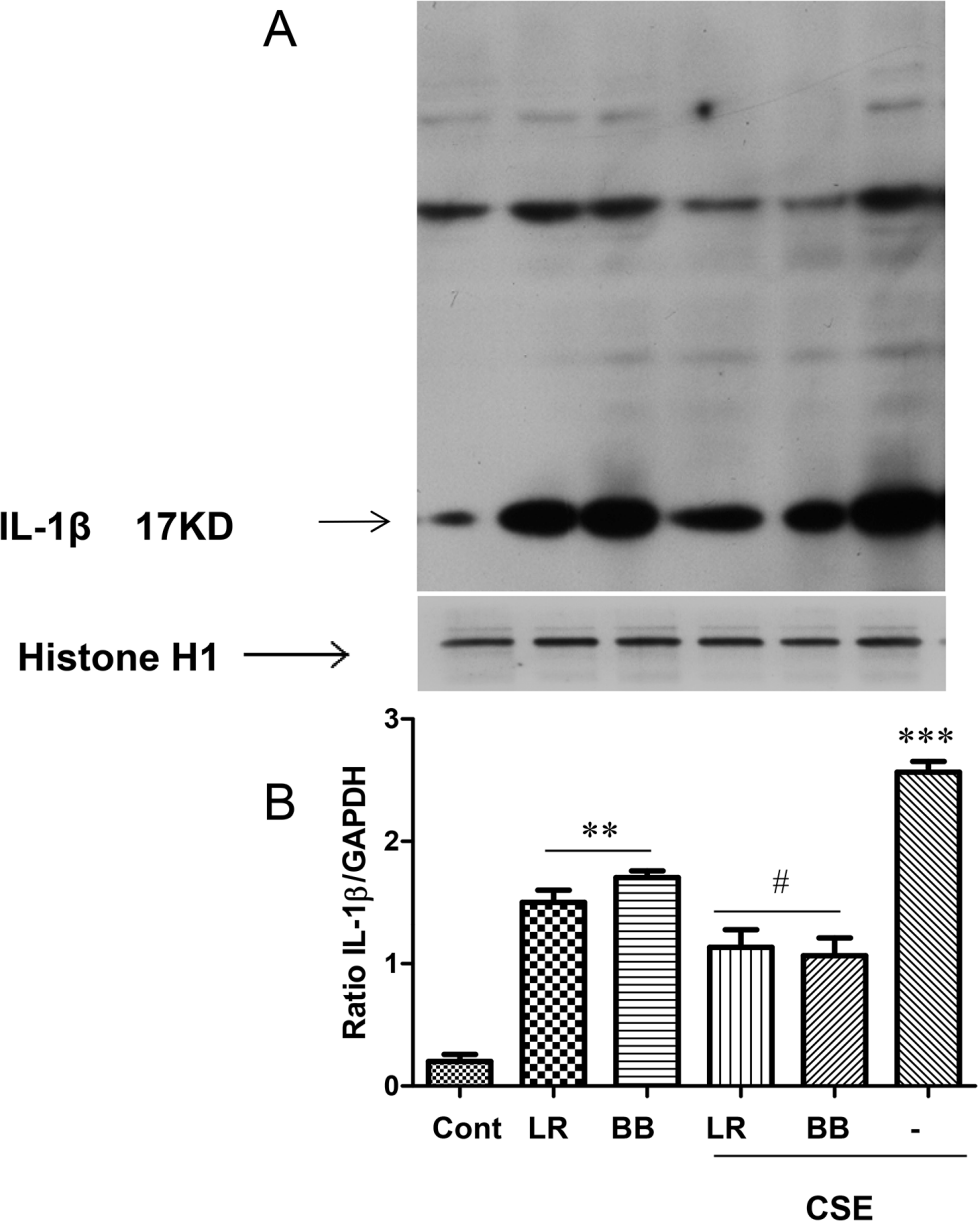
Modulation of IL-1β in THP-1 cells by *L*. *rhamnosus* (LR) and *B*. *breve* (BB) in the presence of cigarette smoke extract (CSE). A representative immunoblot for IL-1β within whole cell extracts of THP-1 cells (5×10^6^) preincubated for 2h with LR and BB (both at 10 bacteria/cell) in the presence or absence of 1.5% CSE for 6hrs (A). Histone H1 (H1) is used as a housekeeping control. Graphical analysis of at least n = 3 independent experiments is presented (B) as the mean±S.E.M. of the ratio to GAPDH expression. *p < 0.05; **p < 0.01 versus control and ^#^p<0.05 versus CSE-stimulated IL-1β expression.

We have previously reported that CSE activation of cells involves IL-1β activation [36]. In Fig 2B we report that CSE-enhanced release of mature IL-1β is suppressed by both *L*. *rhamnosus and B*. *breve*. Western blot analysis of cellular IL-1β expression demonstrated that CSE exposure greatly enhanced the formation of the mature form of IL-1β after 6h (Fig 5, compare lanes 1 and 6). As predicted from the ELISA data (Fig 3B), pre-treatment of cells with *L*. *rhamnosus and B*. *breve* for 2h resulted in a reduction in CSE-induced formation of mature IL-1β. In the absence of CSE-exposure both *L*. *rhamnosus* and *B*. *breve* increased mature IL-1β expression (Fig 5). However, this was not reflected in the ELISA data (Fig 3B) for *L*. *rhamnosus* at the single time point studied suggesting a failure to release IL-1β from the cells in response to *L*. *rhamnosus*.

### Effects of *L*. *rhamnosus* and *B*. *breve* on the HMGB1 expression

**Fig 6:**
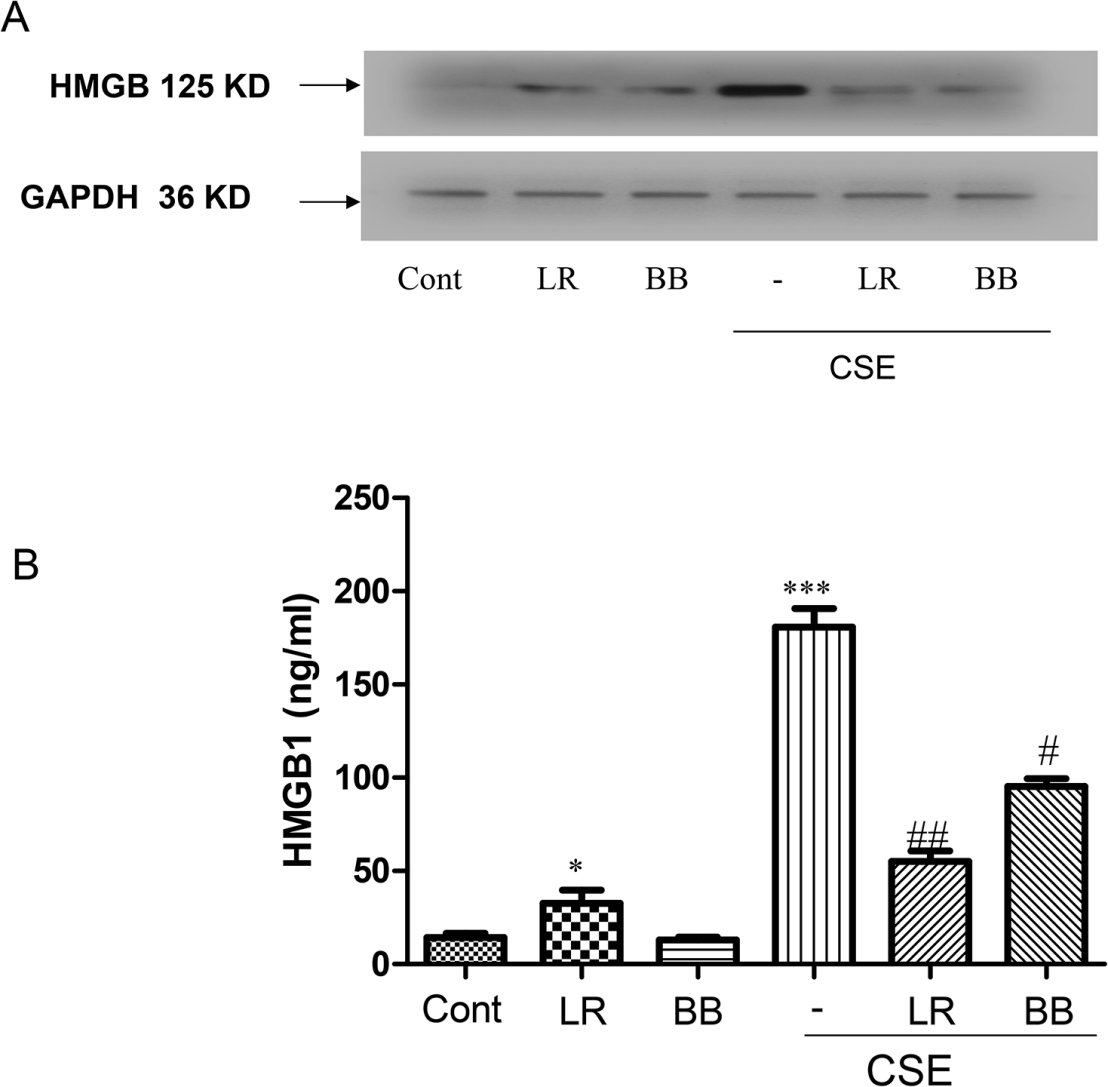
Effects of *L*. *rhamnosus* (LR), *B*. *breve* (BB) and cigarette smoke extract (CSE) on expression and release of HMGB1. Cells were preincubated with LR or BB (both at 10 bacteria/cell) for 2h before stimulation with 1.5% CSE for 5h for analysis of intracellular HMGB1 by Western blotting (A) and 16h for detection of HMGB1 release by ELISA (B). A representative blot of the results from 3 independent experiments is shown with histone H1 (H1) as a housekeeping protein. Data in B are presented as the mean±S.E.M. of 3 independent experiments. *p < 0.05; **p < 0.01 versus control and ^#^p<0.05, ^##^p<0.01 versus CSE-stimulated HMGB1 release.

HMGB1 is also released in response to CSE [41]. We, therefore, determined the effect of *L rhmanosus* and *B*. *breve* on the expression of HMGB1 within cells at 5h and the release of HMGB1 from cells at 16h at baseline and in response to CSE exposure. There was a small but significant increase in basal HMGB1 cell expression (Fig 6A) and release (Fig 6B) following *L*. *rhamnosus* exposure which was not seen with *B*. *breve*. CSE exposure resulted in a highly significant induction of HMGB1 expression and release (Fig 6A & 6B). This was significantly inhibited by 2h pre-treatment with both *L*. *rhamnosus and B*. *breve*. The effect of *L*. *rhamnosus* on CSE-induced HMGB1 expression and release was greater than that seen with *B*. *breve*. This suggests that distinct mechanisms are involved in IL-1β activation by *L*. *rhamnosus and B*. *breve*.

## Discussion

Our data demonstrates that *L*. *rhmanosus* and *B*. *breve* are able to suppress the expression and release of a number of important pro-inflammatory mediators from a CSE-exposed human macrophage-like cell. A similar ability to suppress TLR4 and TLR-9-induced CXCL-8 was also seen. The ability to suppress CS-induced mediators was associated with the attenuation of the key regulatory NF-κB pathway. In contrast, these bacteria enhanced the basal expression of IL-23, IL-10 and CXCL-8 release.

Probiotics are widely used as supplements in dietary products and are defined as live microorganisms that may have the ability to provide a health benefit to the host. Desirable features of a probiotic strain are protective colonization in the intestinal tract and increasing the efficiency of digestion and nutrient absorption and reduced capacity to cause immune responses to deviate towards Th1 and/or Th17 immune responses [42]. Several studies have advocated the potential of probiotics as a therapeutic moiety in asthma and airway infections [38, 43]. However, the rational use of probiotics in airways disease requires a greater knowledge of the effects of these agents on innate immune and inflammatory pathways in the lung.

We used a human macrophage cell line exposed to cigarette smoke as a model to determine the actions of two different beneficial bacterial strains (*L*. *rahmonsus* and *B*. *breve*) on the inflammatory response of human macrophages in COPD. These two bacterial strains were selected as they belong to genera that are widely used in dietary products. Our studies focused on CXCL-8 as this is considered an important chemokine which contributes to COPD pathogenesis by stimulating neutrophil recruitment and promoting their activation and release of proteases [44–46].

Although these strains of bacteria caused increased release of the pro-inflammatory mediators CXCL-8 and IL-23, they also enhanced the release of the anti-inflammatory cytokine IL-10. It is unclear which of these two opposing traits will have a dominating effect in overall macrophage and down-stream T-cell function. Furthermore, there are likely to be distinct mechanisms involved in the pro-inflammatory effects of *L*. *rahmonsus* and *B*. *breve* since the suppression of mediator release was not dose-dependent and occurred equally with both bacteria. Future studies will require the use of gene and/or protein arrays to obtain a better picture of the actions of probiotics on basal macrophage function and testing in a good animal model of COPD. In COPD, the oxidative stress seen after the initial cigarette smoking challenge persists even in subjects who have ceased smoking [47, 48]. This suggests that macrophages from COPD patients will remain in an ‘activated’ state and that modulation of this state is more important than the effects seen in basal unstimulated cells. Ideally, experiments should be performed to examine the effect of probiotics on inflammatory parameters in primary airway or lung macrophages from COPD patients.

We did not find any significant detrimental effects of probiotics on macrophage phagocytosis either on basal or CSE stimulated cells. Defective phagocytosis is an important defect contributing to the pathobiology of COPD [49,50]. It is important, therefore, that these bacteria did not affect this process as this may affect the ability to control infection.

In contrast, we were able to demonstrate a significant inhibition of both *L rhmanosus and B*. *breve* on CSE-activated NF-κB activity. The degree of inhibition of this pathway correlated well with the suppression of inflammatory mediator release. However, since we did not observe complete suppression of mediator release, the involvement of other pathways such as the JNK pathway, which has been previously implicated in probiotic actions [51], cannot be ruled out.

There was a difference in NF-κB activation dependent upon the assay used. SEAP measures functional NF-κB activity after overnight incubation of cells whereas p65 nuclear translocation by Western blot analysis was performed 30 minutes after cell stimulation. NF-κB activity can be enhanced not just by nuclear import but also by post-translational modifications which can markedly affect transcriptional activity without significantly affecting the nuclear levels of p65 [3]. The precise molecular mechanism(s) by which these bacteria are able to activate basal NF-κB activity in macrophages but repress CSE-induced activity is unknown. All bacteria, not just probiotics contain TLR ligands and we hypothesize that it is the panoply of these ligands in probiotics combined with the effect of cigarette smoke on the expression profile of TLRs that give rise to the specific anti-inflammatory effects seen here. Further research in the area should investigate whether these probiotic bacteria may act as partial agonists at TLRs and compete with CSE-induced activation or whether there is cross-talk between cell surface receptors at the level of intracellular co-factors.

The precise mechanism by which IL-1β induced is regulated by CSE and by probiotics unclear. Previous data indicates that nonpathogenic *L*. *rhamnosus* activates the inflammasome leading to caspase-1 activation and IL-1β production [52]. However we were unable to see any induction of IL-1β by *L*. *rhamnosus* or *B*. *breve* from baseline in this study and these probiotics reduced attenuated CSE-induced IL-1β expression. The mechanism by which probiotics to inhibit IL-1β, IL-18 and HMGB1 expression and the possible involvement of inflammasome in the response to TLR3, TLR4 or TLR9 activation and the subsequent effect on IL-1β, may be an area of future research. It is likely that these processes may be more important in exacerbations since there is little evidence for IL-1β and inflammasome activation in stable COPD [53].

Our study here confirms previous work showing that CSE induces the release of HMGB1 [54–57]. HMGB1 expression is increased in COPD patients [58] but the association between HMGB1 expression and DAMPs such as S100 proteins, defensins which are also increased in COPD patients [59], is unclear. We found that the basal release of HMGB1 was increased by CSE treatment and by L. rahmanosus but that both L rhmanosus and B. breve suppress CSE-induced expression and release of HMGB1. The effect of these probiotics on the expression of other innate immune mediators and the mechanism by which they reduce CSE-induced HMGB1 expression needs to be elucidated. The induction of TLR and nucleotide-binding oligomerization domain (NOD)-mediated cross-tolerance may explain these effects of probiotics shown here and this may be a novel therapeutic option in COPD.

Despite the limitations of study, which include the restricted range of probiotics used, the restricted time course for NF-κB analysis and the use of cultured macrophages rather than primary human airway or lung macrophages, we have shown that L. rhamnosus and B. breve inhibit the expression and release of several pro-inflammatory mediators induced by exposure of macrophages to CSE. The precise characterization of the active factor(s) that mediate this effect and its mechanism of action will facilitate the development of new therapeutic strategies for smoking-related airway diseases. Further studies are required to confirm whether L. rhamnosus and B. breve could act as a beneficial probiotics in COPD.
